# Culture harder: use more specimen to increase methicillin-resistant Staphylococcus aureus culture yield relative to PCR

**DOI:** 10.1099/acmi.0.000918.v4

**Published:** 2025-06-03

**Authors:** Arvette E. Mitchell, Arpit P. Patel, Jennifer DiCandilo, Zachary W. Rebollido, Matthew A. Pettengill

**Affiliations:** 1 Department of Pathology and Genomic Medicine, Thomas Jefferson University Hospital, Philadelphia, PA 19107, USA; 2 Department of Pharmacy, Thomas Jefferson University Hospital, Philadelphia, PA 19107, USA

**Keywords:** culture, methicillin-resistant *Staphylococcus aureus *(MRSA) screening, PCR

## Abstract

Methicillin-resistant *Staphylococcus aureus* (MRSA) causes considerable morbidity and mortality in both community-acquired and healthcare-associated infections, but detecting colonization with MRSA has been shown to improve patient outcomes in certain clinical settings. MRSA colonization detection has been carried out in a variety of ways, with molecular assays having superior sensitivity in most studies relative to culture, but culture is disadvantaged in some comparisons by utilization of low specimen volumes. We compared a commercial molecular assay to both low-volume (10 µl) and high-volume (650 µl) cultures and found that increasing the volume utilized for culture led to the detection of 25% more cases than low-volume culture.

## Data Summary

All data from this study are posted as a supplemental data file with the online version of the manuscript.

## Introduction

Methicillin-resistant *Staphylococcus aureus* (MRSA) is a leading cause of both community-acquired and nosocomial infections, causing considerable morbidity and mortality [1,2]. MRSA colonization increases the risk of infection, particularly in healthcare settings, where individuals are more likely to be vulnerable or immunocompromised. Detecting MRSA and implementing decolonization or targeted therapy can reduce poor patient outcomes in a variety of clinical settings and also lead to overall cost savings for the healthcare system [3].

There are a variety of ways in which the evaluation of MRSA colonization can be carried out. Studies have demonstrated significant differences obtained at preanalytical steps based on different types of swabs [4,5] and different upper respiratory or other anatomical sites evaluated for colonization [6]. The primary methods of evaluation of collected material are culture and PCR with many different options for each. Culture may be carried out on conventional media such as trypticase soy agar with 5% sheep’s blood or Columbia nalidixic acid, looking for the presence of characteristic β-hemolytic colonies by using MALDI-TOF or biochemical or latex agglutination methods. Alternatively, the use of chromogenic agar [7] has become increasingly common wherein cultures are screened based on the appearance of colonies of a certain colour that varies by manufacturer, and this form of culture is readily adapted to reading by total laboratory automation systems [8]. Both conventional culture and chromogenic agar culture may be preceded by enrichment culture where the specimen is first inoculated into broth for some period of time before inoculating from the enrichment broth to solid agar [5] – the enrichment step improves sensitivity but delays result reporting. The use of molecular methods, primarily PCR, to directly test the collected material for the presence of MRSA is becoming increasingly common. PCR assays are more sensitive than culture, even enrichment culture, and can be much faster than the culture-based methods depending on the laboratory workflow.

There have been many studies comparing the sensitivity of culture-based methods and PCR for MRSA detection from nares swabs, with culture being 58–85% sensitive relative to PCR [5,6, 9,16]. Discrepant cases that are PCR-positive and culture-negative may occur for a variety of reasons. Culture requires viable organisms, whereas PCR does not; it only requires intact DNA for the assay targets. A patient may have recently been colonized with viable MRSA, but prior to sample collection, the organism died due to competition or antibiotic therapy. Nares colonization always likely includes both viable and non-viable MRSA when the former is present, giving an advantage to PCR which will detect intact DNA from non-viable organisms that would not grow in culture. At least one previous study found a significant correlation between improved performance of PCR relative to culture in patients treated with MRSA-active antibiotics [15]. However, vancomycin therapy has specificallybeen shown not to alter nares MRSA colonization rates [17,18]. Genetic modification, such as *mecA* dropout [12], may also leave an organism detectable as MRSA by PCR if the assay targets *SCCmec* cassette targets as proxy for *mecA*, while being phenotypically susceptible to methicillin/oxacillin and not detectable (Chromagar) or detected as Methicllin-susceptible *Staphylococcus aureus* (MSSA) (traditional culture and susceptibility testing).

The volume of specimen analysed may also lead to differences in detection rates for culture compared with PCR. Commonly used commercial MRSA PCR assays are loaded with 200–300 µl of specimen [5,19], whereas cultures use variable quantities of specimen, but generally far lower than is processed in molecular assays. Automated systems for plate streaking (WASPlab, BD Kiestra) have been studied using 30 µl [8,20] or even just 10 µl of sample [21,23].

We sought to evaluate two elements that have been poorly characterized in the past: the impact of sample volume analysed and prior treatment with antibiotics, on culture sensitivity relative to PCR for nares MRSA colonization.

## Methods

Luminex ARIES MRSA PCR was performed for clinical purposes from 507 patient samples collected using a 1 ml Eswab (collected from both nares) according to the manufacturer’s instructions (300 µl processed and 200 µl loaded into the assay cartridge). Remnant specimen was plated to Becton Dickinson BBL MRSA CHROMagar at 10 µl and 650 µl (650 µl was the maximum that was consistently available after using 300 µl for PCR and 10 µl for low-volume culture (LVCx); for the higher volume culture, the fluid specimen was allowed to soak into the agar for 15 min before flipping the plates). After the specimen was inoculated to the media, the plate was incubated at 35℃ in ambient air for 20–24 h; the presence of mauve colonies was accepted as the presence of MRSA, although specimens that had mauve colonies but were MRSA PCR negative were investigated further. Colonies from culture-positive but PCR-negative specimens were confirmed as MRSA using MALDI-TOF MS (Bruker) and cefoxitin disc diffusion testing. However, no specimen remained for discrepant analysis of PCR-positive but culture-negative results as the specimen volume was exhausted. For the purposes of the study, these were accepted as true positive for comparison. Results were also correlated to lower respiratory tract cultures if performed within +/−7 days of PCR collection.

We also reviewed the medication history of each patient with positive results from at least one of the MRSA detection assays and recorded the utilization of any MRSA-active antibiotics within 7 days prior to the collection of the nares PCR specimen. We considered MRSA-active agents as vancomycin, daptomycin, doxycycline, trimethoprim-sulfamethoxazole (TMP-SMX), levofloxacin, ciprofloxacin and clindamycin.

## Results

A total of 507 specimens from 501 unique patients were evaluated across the following three test conditions: PCR, high-volume culture (HVCx) and LVCx. A total of 93 samples were positive for at least one MRSA detection assay, 29 were positive for PCR only and 6 were positive for culture only. The per cent sensitivity overall was 94% for PCR, 69% for HVCx and 55% for LVCx (Table 1). A total of 64 specimens were positive for HVCx compared with 51 for LVCx; thus, a higher volume culture increased the sensitivity of culture by 25%. We also compared assay results by category if the specimen was received within +/−7 of a lower respiratory tract culture [sputum or bronchoalveolar lavage (BAL)], for which 98 samples had such results for comparison (the majority of which, 69/98, were received within +/−1 day of the MRSA nares PCR order). Our lab rules out MRSA in lower respiratory tract cultures (by screening any β-hemolytic colonies for MRSA). Among specimens with both lower respiratory tract cultures and MRSA PCR ordered, there were 12 specimens (from 12 patients) from which MRSA was recovered in the lower respiratory tract culture. Although this subset is small in sample size, it is interesting to note that there was no disagreement between the MRSA detection assays amongst this group: nine detected by all assays, but three detected by none. The three lower respiratory tract specimens that were culture-positive but not detected by nares screening were a sputum collected 5 days after nares PCR was ordered, another sputum collected 1 day prior to nares PCR being ordered and a patient with two BAL cultures, both of which grew MRSA and were collected on the same day the nares screen PCR was ordered.

**Table 1.** Results categorized by negativity (−) or positivity (+) of PCR, HVCx and LVCx

Group	Total	PCR −HVCx −LVCx −	PCR +HVCx + LVCx +	PCR +HVCx −LVCx −	PCR +HVCx +LVCx −	PCR −HVCx +LVCx −	PCR −HVCx +LVCx +	PCR sens%	HVCx sens%	LVCx sense%
all samples	507	414	50	29	8	5	1	94 %	69 %	55 %
no LRT culture	409	337	38	24	6	3	1	94 %	67 %	54 %
LRT culture negative for MRSA	86	74	3	5	2	2	0	83 %	58 %	25 %
LRT culture positive for MRS	12	3	9	0	0	0	0	100 %	100 %	100 %

Sensitivity calculated compared only to screening test result composite, not to clinical sputum/BAL culture. No samples were positive only by LVCx.

Cx, culture; HVCx, high-volume culture (650 µL); LRT, lower respiratory tract; LVCx, low-volume culture, (10 µL); sens%, per cent sensitivity relative to aggregate.

Utilization of MRSA-active antibiotics within 7 days prior to nares PCR specimen collection did not correlate with PCR-positive, culture-negative screens; however, this was essentially limited to an evaluation of vancomycin. Among 93 patients evaluated, 48 had received vancomycin, whereas only 3 had received clindamycin, 2 each levofloxacin, doxycycline or TMP-SMX and 1 each daptomycin or ciprofloxacin. In fact, the trend (not statistically significant, student’s t-test) was in the opposite direction of what we expected – a higher percentage of culture-positive specimens (63%, 40/64) were from patients with recent MRSA-active antibiotic administration, compared with PCR-positive but culture-negative specimens (41%, 12/29).

## Discussion

Our findings are consistent with previous studies in demonstrating that culture-based methods are less sensitive than nucleic-acid-amplification-based methods such as PCR for the detection of nares colonization with MRSA [5,6, 9,16]. Additionally, our review of medication history is consistent with prior work that found that systemic administration of vancomycin does not impact nares colonization with MRSA [17,18]. Interestingly, we found that results across PCR and LVCx/HVCx were perfectly correlated among the 12 patients with lower respiratory tract cultures that were positive for MRSA – detected by all assay formats for 9/12 patients, but negative in all for 3/12 patients. This rate of negative MRSA colonization screening among patients with MRSA-positive lower respiratory tract cultures is consistent with what was observed in a larger study [24].

Our work adds new information regarding the degree to which the volume of specimens plated for culture impacts the sensitivity of culture. In our study conditions (1 ml Eswab collection), a 650 µl culture inoculation detected 25% more positive cases than 10 µl culture inoculation, the latter volume being one that is commonly used with modern automation platforms [21,23]. We believe our findings provide compelling evidence that if culture must be employed for nares MRSA detection instead of PCR, for financial or technical considerations, labs should *culture harder* by using as much volume from the collection device as feasible.

## Supplementary material

10.1099/acmi.0.000918.v4 Uncited Fig. S1.

## Abbreviations

HVCx: high-volume culture
LVCx: low-volume culture
MRSA: methicillin-resistant *Staphylococcus aureus*
TMP-SMX: trimethoprim-sulfamethoxazole

## References

[1] Hanberger H, Walther S, Leone M, Barie PS, Rello J (2011). Increased mortality associated with methicillin-resistant *Staphylococcus aureus* (MRSA) infection in the intensive care unit: results from the EPIC II study. Int J Antimicrob Agents.

[2] Delaney JA, Schneider-Lindner V, Brassard P, Suissa S (2008). Mortality after infection with methicillin-resistant *Staphylococcus aureus* (MRSA) diagnosed in the community. BMC Med.

[3] Meng L, Pourali S, Hitchcock MM, Ha DR, Mui E (2021). Discontinuation patterns and cost avoidance of a pharmacist-driven methicillin-resistant *Staphylococcus aureus* nasal polymerase chain reaction testing protocol for de-escalation of empiric vancomycin for suspected pneumonia. Open Forum Infect Dis.

[4] Dalpke AH, Hofko M, Stock C, Zimmermann S (2014). Evaluation of the BD Max MRSA XT assay for use with different swab types. J Clin Microbiol.

[5] Yarbrough ML, Warren DK, Allen K, Burkholder D, Daum R (2018). Multicenter evaluation of the Xpert MRSA NxG assay for detection of methicillin-resistant *Staphylococcus aureus* in nasal swabs. J Clin Microbiol.

[6] Molan A, Nulsen M, Thomas G (2013). Methicillin-resistant *Staphylococcus aureus* (MRSA): isolation from nasal and throat swabs transported in liquid or semisolid media; identification by PCR compared with culture. N Z J Med Lab Sci.

[7] Perry JD (2017). A decade of development of chromogenic culture media for clinical microbiology in an era of molecular diagnostics. Clin Microbiol Rev.

[8] McElvania E, Mindel S, Lemstra J, Brands K, Patel P (2024). Automated detection of methicillin-resistant *Staphylococcus aureus* with the MRSA CHROM imaging application on BD Kiestra total lab automation system. J Clin Microbiol.

[9] Hiermandi N, Foster C, Purnell K, Dunn J, Campbell J (2023). MRSA PCR improves sensitivity of detection of colonization in neonates. Antimicrob Steward Healthc Epidemiol.

[10] Luteijn JM, Hubben GAA, Pechlivanoglou P, Bonten MJ, Postma MJ (2011). Diagnostic accuracy of culture-based and PCR-based detection tests for methicillin-resistant *Staphylococcus aureus*: a meta-analysis. Clin Microbiol Infect.

[11] Nielsen XC, Madsen TV, Engberg J (2017). Evaluation of Xpert MRSA Gen 3 and BD MAX MRSA XT for meticillin-resistant *Staphylococcus aureus* screening in a routine diagnostic setting in a low-prevalence area. J Med Microbiol.

[12] Snyder JW, Munier GK, Johnson CL (2010). Comparison of the BD GeneOhm methicillin-resistant *Staphylococcus aureus* (MRSA) PCR assay to culture by use of BBL CHROMagar MRSA for detection of MRSA in nasal surveillance cultures from intensive care unit patients. J Clin Microbiol.

[13] Tansarli GS, LeBlanc L, Auld DB, Chapin KC (2020). Diagnostic accuracy of presurgical *Staphylococcus aureus* PCR assay compared with culture and post-PCR implementation surgical site infection rates. J Mol Diagn.

[14] Wolk DM, Marx JL, Dominguez L, Driscoll D, Schifman RB (2009). Comparison of MRSASelect agar, CHROMagar methicillin-resistant *Staphylococcus aureus* (MRSA) medium, and Xpert MRSA PCR for detection of MRSA in nares: diagnostic accuracy for surveillance samples with various bacterial densities. J Clin Microbiol.

[15] Shenoy ES, Noubary F, Kim J, Rosenberg ES, Cotter JA (2014). Concordance of PCR and culture from nasal swabs for detection of methicillin-resistant *Staphylococcus aureus* in a setting of concurrent antistaphylococcal antibiotics. J Clin Microbiol.

[16] Peterson LR, Liesenfeld O, Woods CW, Allen SD, Pombo D (2010). Multicenter evaluation of the LightCycler methicillin-resistant *Staphylococcus aureus* (MRSA) advanced test as a rapid method for detection of MRSA in nasal surveillance swabs. J Clin Microbiol.

[17] Yu VL, Goetz A, Wagener M, Smith PB, Rihs JD (1986). *Staphylococcus aureus* nasal carriage and infection in patients on hemodialysis. Efficacy of antibiotic prophylaxis. *N Engl J Med*.

[18] Carr AL, Daley MJ, Givens Merkel K, Rose DT (2018). Clinical utility of methicillin-resistant *Staphylococcus aureus* nasal screening for antimicrobial stewardship: a review of current literature. Pharmacotherapy.

[19] Silbert S, Kubasek C, Galambo F, Vendrone E, Widen R (2015). Evaluation of BD Max StaphSR and BD Max MRSA XT assays using ESwab-collected specimens. J Clin Microbiol.

[20] Herrmann M, Petit C, Dawson A, Biechele J, Halfmann A (2013). Methicillin-resistant *Staphylococcus aureus* in Saarland, Germany: a statewide admission prevalence screening study. PLoS One.

[21] Burckhardt I, Horner S, Burckhardt F, Zimmermann S (2018). Detection of MRSA in nasal swabs-marked reduction of time to report for negative reports by substituting classical manual workflow with total lab automation. Eur J Clin Microbiol Infect Dis.

[22] Cheng CWR, Ong CH, Chan DSG (2020). Impact of BD Kiestra InoqulA streaking patterns on colony isolation and turnaround time of methicillin-resistant *Staphylococcus aureus* and carbapenem-resistant Enterobacterale surveillance samples. Clin Microbiol Infect.

[23] Gammel N, Ross TL, Lewis S, Olson M, Henciak S (2021). Comparison of an automated plate assessment system (APAS Independence) and artificial intelligence (AI) to manual plate reading of methicillin-resistant and methicillin-susceptible *Staphylococcus aureus* CHROMagar surveillance cultures. J Clin Microbiol.

[24] Turner SC, Seligson ND, Parag B, Shea KM, Hobbs ALV (2021). Evaluation of the timing of MRSA PCR nasal screening: how long can a negative assay be used to rule out MRSA-positive respiratory cultures?. Am J Health Syst Pharm.

