# Erratum: Culture harder: use more specimen to increase methicillin-resistant Staphylococcus aureus culture yield relative to PCR

**DOI:** 10.1099/acmi.0.001063

**Published:** 2025-06-30

**Authors:** Arvette E. Mitchell, Arpit P. Patel, Jennifer DiCandilo, Zachary W. Rebollido, Matthew A. Pettengill

**Affiliations:** 1 Department of Pathology and Genomic Medicine, Thomas Jefferson University Hospital, Philadelphia, PA 19107, USA; 2 Department of Pharmacy, Thomas Jefferson University Hospital, Philadelphia, PA 19107, USA

During the copyediting stage of production an error was introduced to the title of this article. This resulted in misspelling of the word ‘specimen’ in the title of the published version of the record. The correct title is ‘Culture harder: use more specimen to increase methicillin-resistant Staphylococcus aureus culture yield relative to PCR.’

Previously the title was ‘Culture harder: use more specimens to increase methicillinresistant Staphylococcus aureus culture yield relative to PCR.’

The version of record was updated to reflect this correction.

We apologise for the inconvenience.

